# VAMPr: VAriant Mapping and Prediction of antibiotic resistance via explainable features and machine learning

**DOI:** 10.1371/journal.pcbi.1007511

**Published:** 2020-01-13

**Authors:** Jiwoong Kim, David E. Greenberg, Reed Pifer, Shuang Jiang, Guanghua Xiao, Samuel A. Shelburne, Andrew Koh, Yang Xie, Xiaowei Zhan

**Affiliations:** 1 Quantitative Biomedical Research Center, Department of Population and Data Sciences, University of Texas Southwestern Medical Center, Dallas, Texas, United States of America; 2 Department of Internal Medicine, University of Texas Southwestern Medical Center, Dallas, Texas, United States of America; 3 Department of Microbiology, University of Texas Southwestern Medical Center, Dallas, Texas, United States of America; 4 Department of Statistical Science, Southern Methodist University, Dallas, TX, United States of America; 5 Harold C. Simmons Cancer Center, University of Texas Southwestern Medical Center, Dallas, Texas, United States of America; 6 Department of Bioinformatics, University of Texas Southwestern Medical Center, Dallas, Texas, United States of America; 7 Department of Infectious Diseases and Genomic Medicine, University of Texas MD Anderson Cancer Center, Houston, Texas, United States of America; 8 Department of Pediatrics, University of Texas Southwestern Medical Center, Dallas, Texas, United States of America; 9 Center for Genetics of Host Defense, University of Texas Southwestern Medical Center, Dallas, Texas, United States of America; DAL, CANADA

## Abstract

Antimicrobial resistance (AMR) is an increasing threat to public health. Current methods of determining AMR rely on inefficient phenotypic approaches, and there remains incomplete understanding of AMR mechanisms for many pathogen-antimicrobial combinations. Given the rapid, ongoing increase in availability of high-density genomic data for a diverse array of bacteria, development of algorithms that could utilize genomic information to predict phenotype could both be useful clinically and assist with discovery of heretofore unrecognized AMR pathways. To facilitate understanding of the connections between DNA variation and phenotypic AMR, we developed a new bioinformatics tool, variant mapping and prediction of antibiotic resistance (VAMPr), to (1) derive gene ortholog-based sequence features for protein variants; (2) interrogate these explainable gene-level variants for their known or novel associations with AMR; and (3) build accurate models to predict AMR based on whole genome sequencing data. We curated the publicly available sequencing data for 3,393 bacterial isolates from 9 species that contained AMR phenotypes for 29 antibiotics. We detected 14,615 variant genotypes and built 93 association and prediction models. The association models confirmed known genetic antibiotic resistance mechanisms, such as *blaKPC* and carbapenem resistance consistent with the accurate nature of our approach. The prediction models achieved high accuracies (mean accuracy of 91.1% for all antibiotic-pathogen combinations) internally through nested cross validation and were also validated using external clinical datasets. The VAMPr variant detection method, association and prediction models will be valuable tools for AMR research for basic scientists with potential for clinical applicability.

## Author summary

Antimicrobial resistance (AMR) is a global health threat. The current method to determine AMR is inefficient and complete understanding of the mechanisms of AMR is lacking. With the increased feasibility of sequencing bacterial genomes, it is now easier, faster and cheaper to have genomic insights into AMR. In this manuscript, we propose a novel bioinformatic tool for variant mapping and prediction of antibiotic resistance (VAMPr). We curated 3,393 bacterial genomes from 9 bacterial species that contained AMR phenotypes for 29 antibiotics. We used protein orthology and detected 14,615 variants. Combined with AMR phenotypes, we built 93 association and prediction models. The association model confirms known genetic AMR mechanisms, and the prediction models achieved high accuracies. Together, our work will be valuable for AMR research for basic scientists with the potential for clinical applicability.

## Introduction

Antimicrobial resistance (AMR) is an urgent worldwide threat [1]. Decreased efficacy of antibiotics can lead to prolonged hospitalization and increased mortality [2]. Current phenotypic methods for determining whether an isolate is sensitive or resistant to a particular antibiotic can, in some instances, take days resulting in delays in providing effective therapy [3]. Targeted methods for AMR determination, such as PCR, are limited in that they identify only a subset of resistant genes and therefore do not provide a full explanation for a particular resistance phenotype [4].

Next-generation sequencing (NGS) technology enabling whole genome sequencing (WGS) of bacterial isolates is now both inexpensive and widely-used [5]. A recent review illustrates how this promising technology could enable genome-based prediction for antibiotic resistance [6]. We have previously shown that NGS can identify AMR determinants for a limited number of β-lactam antimicrobials using a rule-based method and that genotype correlated well with classic phenotypic testing [7]. However, that study focused on a narrow set of both antibiotics and pathogens because the links between genotype and phenotype are relatively well understood for those antibiotic/pathogen combinations. To build prediction models for a broader spectrum of antimicrobials, it is necessary to use model-based based methods to study the complex relationship among resistance loci. For example, other groups have utilized NGS data to identify the presence of genes or short nucleotide sequences that confer resistance in a variety of pathogens using k-nn or adaBoost algorithms [8–10]. However, these studies have not taken advantage of gene orthology features. In addition, mechanisms of AMR for many pathogen-antibiotic combinations are not well delineated which hinders the understanding of genotypic-phenotypic relationships. Therefore, we sought to utilize large bacterial data collections in order to develop novel approaches (association and prediction models) to characterize explainable genetic features that correlate with antimicrobial resistance.

## Results

### VAMPr: A novel bioinformatics resource to study microbial resistance

**Fig 1:**
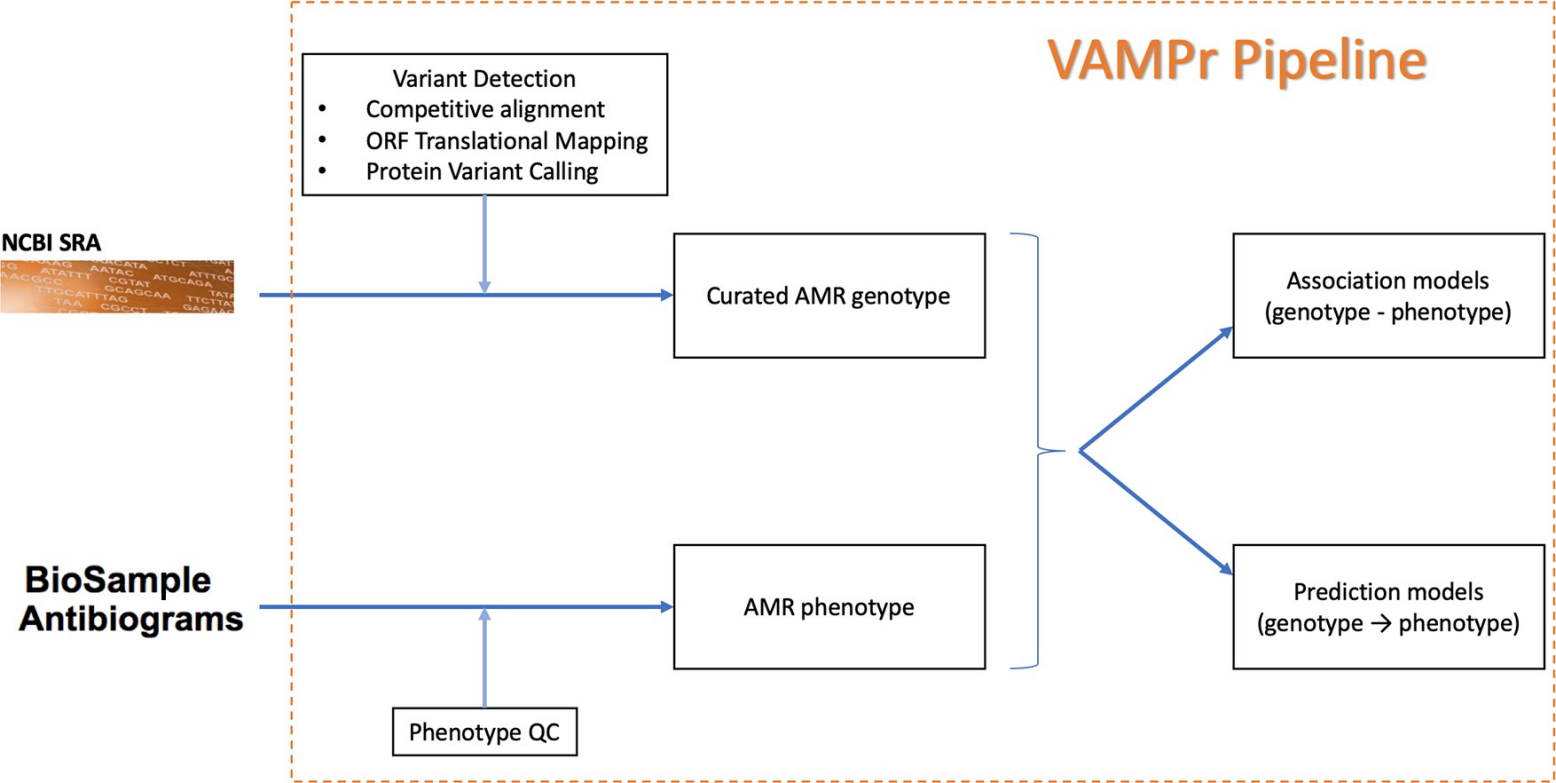
Overview of the VAMPr workflow. The VAMPr pipeline processed sequence data from the NCBI Short Read Achieve (SRA) and NCBI BioSample Antibiograms for phenotypes. The curated AMR genotypes and AMR phenotypes were used to create both association and prediction models.

In order to more fully explore genotypic prediction of antibiotic resistance and build upon our previous efforts, we have developed novel methods for utilizing NGS data to better 1) characterize amino-acid based variant features, 2) expand the knowledge base of genetic associations with AMR, and 3) construct accurate prediction models for determining phenotypic resistance from NGS data in a broad array of pathogen-antibiotic combinations. We developed a novel bioinformatics resource, **VA**riant **M**apping and **P**rediction of antibiotic **r**esistance, VAMPr (**Fig 1**). It was built utilizing a large dataset of bacterial genomes from the NCBI Sequence Read Archive (SRA) along with paired antibiotic susceptibility data from the NCBI BioSample Antibiogram. VAMPr utilizes two different approaches, association models and prediction models, to assess genotype-phenotype relationships. In the association analysis, data-driven association models utilizing a gene ortholog approach were constructed. This allowed for unbiased screening of genotype and phenotype across a broad array of bacterial isolates. In the prediction analysis, we utilized a machine learning algorithm to develop prediction models that take NGS data and predict resistance for every pathogen-drug combination. These approaches not only confirmed known genetic mechanisms of antibacterial resistance, but also identified potentially novel or underreported correlates of resistance.

We depicted the VAMPr workflow in **Fig 1**. First, we downloaded publicly available bacterial genomes from the NCBI Short Read Archive (SRA) and paired antibiotic susceptibility data from the NCBI BioSample Antibiograms project. In order to identify bacterial genetic variants, we performed *de novo* assembly and aligned the assembled scaffolds to a curated Antimicrobial Resistance (AMR) KEGG orthology database (KO) [11]. Through this process KO-based sequence variants were identified. CLSI breakpoints were used to determine the antibiotic phenotype (sensitive versus resistant; isolates with intermediate susceptibility were not included for analysis) [12]. Finally, factoring both genetic variants and antibiotic resistance phenotypes, association and prediction models were constructed. These models are available to the research community through our website (see **Data Access**).

### Construction of NCBI datasets of curated genotypes and phenotypes

**Fig 2:**
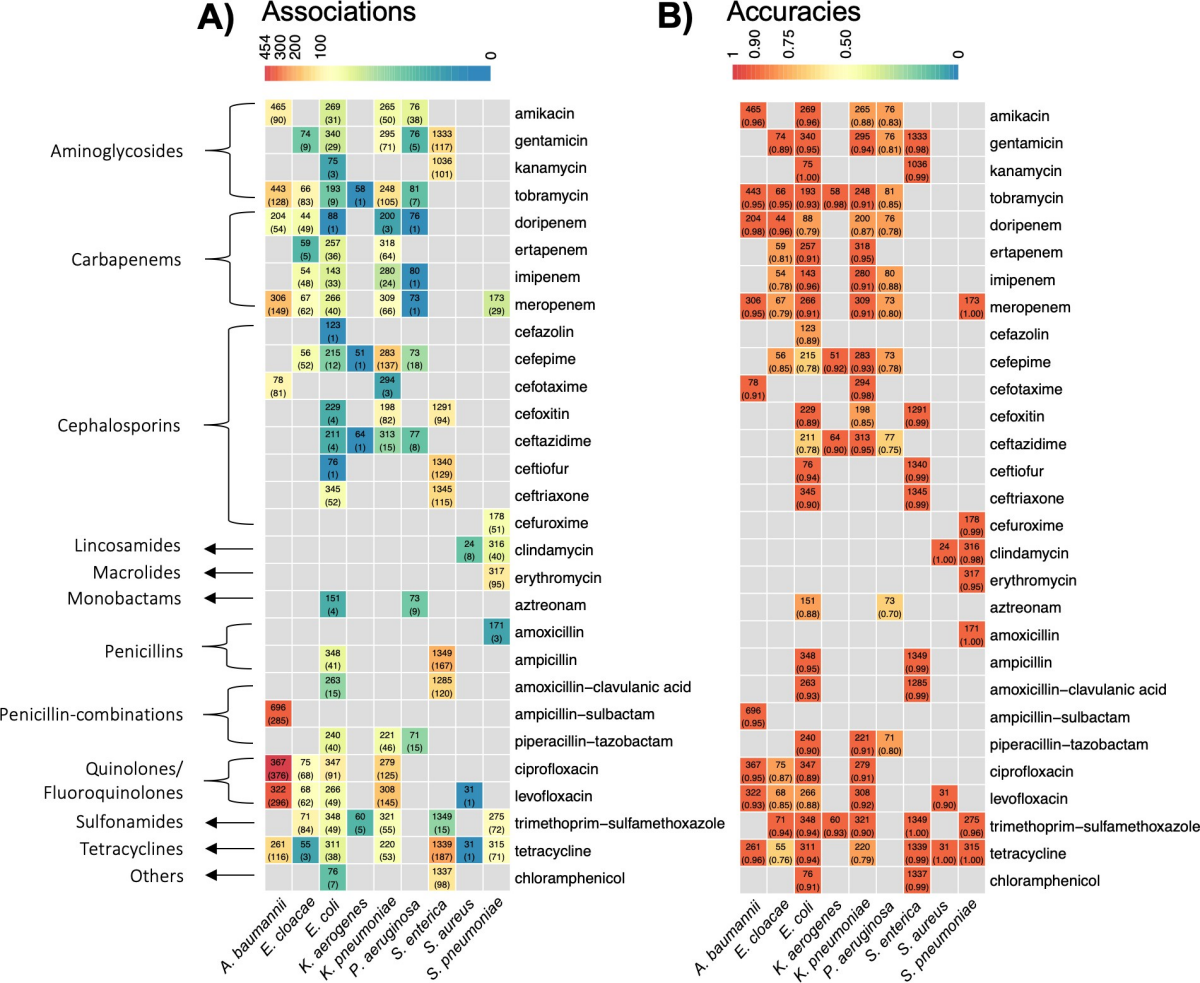
Summary of significant variant associations and prediction accuracies from 93 species-antibiotics combinations. Both heatmaps display the counts of curated isolates by the combination of 9 bacterial species and 29 antibiotics from 13 drug categories. The boxes without a number indicates that no isolates were available for this particular bacterial species and antibiotic combination. A) the color of the boxes indicates the number of gene-antibiotic resistance associations with FDR adjusted p-values <0.05 from VAMPr association models, and the actual numbers are shown within the parenthesis; B) the color indicates cross-validated prediction accuracies from VAMPr prediction models, and the accuracies are shown within the parenthesis.

Focusing on the isolates reported in the NCBI Antibiogram database, we retrieved 4,515 bacterial whole genome sequence datasets (Illumina platform) from NCBI SRA and their antimicrobial resistance phenotypes from NCBI BioSample Antibiograms project. Sequence reads were *de novo* assembled and aligned to Multi Locus Sequence Typing (MLST) databases to validate reported bacterial species identification [13]. 1,100 isolates were excluded from analysis because of inaccurate species identification. Our final analysis cohort included 3,393 isolates representing 9 species: *Salmonella enterica* (1349 isolates), *Acinetobacter baumannii* (772), *Escherichia coli* (350), *Klebsiella pneumoniae* (344), *Streptococcus pneumoniae* (317), *Pseudomonas aeruginosa* (83), *Enterobacter cloacae* (79), *Klebsiella aerogenes* (68), and *Staphylococcus aureus* (31). A total of 38,871 MIC (minimal inhibitory concentration, the lowest antibiotic concentration to inhibit bacterial growth) values were reported for 29 different antibiotics (**S1 Table and S2 Table**). In total, there were 38,248 individual pathogen-drug data points identified (**Fig 2A**).

After curation, we analyzed isolates with *de novo* assembled genome and MIC values, and this dataset included 93 species/antibiotic combinations for building association and prediction models (detailed in next 3 sections). The fraction of resistant isolates for any given bacteria and antibiotic varied greatly (the median fraction of resistant isolates was 50.0%). As an example, for *S*. *enterica* and trimethoprim-sulfamethoxazole, the fraction of resistant isolates was 0.6% while for *K*. *pneumoniae* and cefazolin, the fraction of resistant isolates was 97.3%. This dataset was used in both the association and prediction models.

### Characterization of explainable AMR sequence variants

We curated a list of 537 Antimicrobial Resistance (AMR) KEGG ortholog (KO) genes (**S3 Table**) and then identified the corresponding UniRef protein sequences (a total of 298,760 sequences). Protein sequences were then clustered (using a minimal sequence similarity of 0.7). This resulted in 96,462 KO gene clusters to serve as a reference AMR protein sequence database. Next, we analyzed 3,393 *de novo* assembled genomes, identified the gene locations on the assembled genomes, and aligned the gene sequences to the reference AMR protein sequence database. Based on the alignment results and stringent filtering, we can identify AMR genes for each isolate. Finally, the AMR genes were examined for the presence of mutations (e.g. amino acid substitutions) using multiple sequence alignment software. We nominated an identifier format to represent the sequences. For example, K01990.129|290|TN|ID indicates that the 129^th^ cluster of K01990 KO gene has mutation starting from its 290^th^ amino acid from threonine (T) and asparagine (N) to isoleucine (I) and aspartic acid (D).

### Association models between sequence variants and antibiotic resistance phenotypes retain accuracy

**Fig 3:**
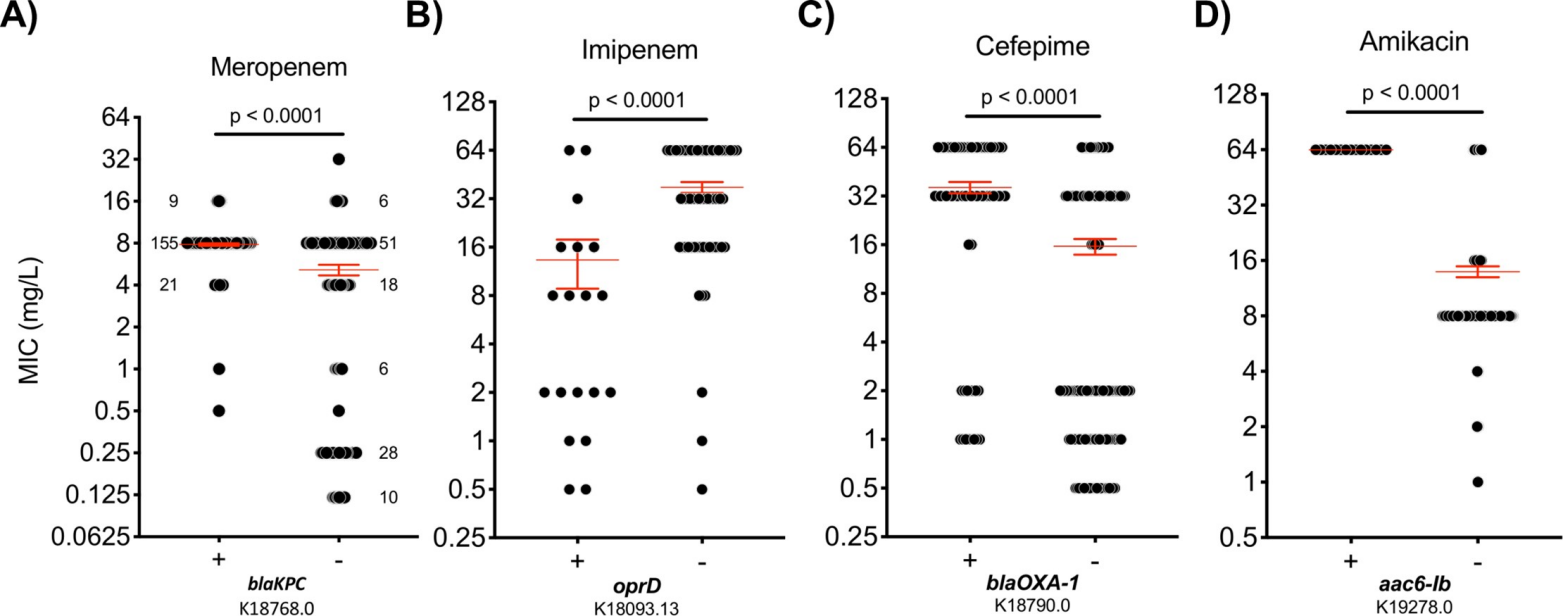
Examples of variant-phenotype relationships determined by the association models. (A) K18768.0 indicates blaKPC, the *K*. *pneumoniae* carbapenemase. The presence of blaKPC is associated with resistance to ceftazidime in *K*. *pneumoniae* as shown. The numbers in the plots represent the frequency of certain MIC (minimal inhibitory concentration) values. Numbers in the plot represent total number of isolates with the given MIC value. (B) K18093.13 is oprD, an imipenem/basic amino acid-specific outer membrane pore; absence of oprD is associated with resistance to imipenem in *P*. *aeruginosa*. (C) K18790.0 represents blaOXA-1, the beta-lactamase class D OXA-1. Its presence is associated with resistance to cefepime in *E*. *coli*. (D) K19278.0 is aac6-lb gene. The presence of this variant is associated with amikacin resistance in *A*. *baumannii*. The “+” and “-”sign in the X-axis represent whether the wild-type gene exists or not. The red horizontal lines mark the mean and standard error of the groupwise MIC measurements. Each gray dot represents an MIC value. P-values are calculated based on Fisher’s exact test. MIC: minimal inhibitory concentration.

We interrogated the strength of the association model between genetic variants and antibiotic susceptibility phenotypes for each bacterial species and antibiotic combination. For a number of pathogen-antibiotic pairs, the association model accuracy was greater than 95% (ranged from 69.6% for *Pseudomonas aeruginosa*-aztreonam to 100.0% for *S*. *pneumoniae*-tetracycline; mean accuracy was 91.1%) (**Fig 2B**). Utilizing contingency tables of variant carrying status and resistance phenotypes with the appropriate statistical analysis (odds ratios and p-values from Fisher’s exact tests), we examined a subset of 5,359 associations with false discovery rates less than 0.05. In many instances, a significantly strong association confirmed an expected antibiotic resistance mechanism (**Fig 3**). For example, the sequence variant K18768.0 represents β-lactamase (Bla) encoding gene *bla*_*KPC*_, the *K*. *pneumoniae* carbapenemase whose presence is significantly associated with resistance to meropenem in *K*. *pneumoniae* (P-value <0.0001) [10](**Fig 3A**). Variant K18093.13 is *oprD*, a major porin responsible for uptake of carbapenems in *Pseudomonas* [14]. Loss of porin activity by *Pseudomonas* is well known to result in carbapenem resistance [15] in this pathogen, and absence of wild-type *oprD* is strongly associated with imipenem resistance (P-value <0.0001) (**Fig 3B**). Other examples (*OXA-1* and *aac-(6’)-lb*) of strong associations are illustrated in **Fig 3C** and **3D**.

### Antibiotic resistance prediction models developed utilizing machine learning

Our association studies demonstrated the accuracy of our genotypic approach for known AMR elements. To begin to explore the capacity of our approach to take sequence data and generate robust prediction, we first developed 93 different prediction models using the VAMPr pipeline. The most promising prediction models were based on an extreme gradient boosting tree algorithm and all hyper-parameters were fine-tuned in the inner 5-fold cross validation. Other prediction models (e.g. elastic net [16], support vector machines, 3-layer neural network, and adaptive boosting) were evaluated but did not exhibit superior prediction performances (**S4 Fig**). For all models, we used nested cross validation to report prediction performance metrics (**Table 1, S4 Table)**. Among 93 models, half had prediction accuracies greater than 90%. The pathogen-antibiotic combinations that displayed the highest accuracy were for *S*. *pneumoniae* (clindamycin (100.0%), meropenem (100.0%), and tetracycline (100.0%), and *E*. *coli* and kanamycin (100.0%). 11 prediction models for *S*. *enterica* had very high accuracies (minimal prediction accuracy is 98.0%) likely due to the larger dataset of *S*. *enterica* isolates. A similar trend was also seen in the performance of the models for *A*. *baumannii*.

**Table 1:** Prediction metrics for 32 VAMPr prediction models. Among 93 prediction models, we listed the top 32 models that have the mean prediction accuracies higher than 95%. The isolate and variant counts derived from sequencing were used to build the prediction model using gradient boosting tree algorithms. The accuracy is reported using nested cross validation approach. The 10-fold outer cross validation were used to report accuracy and the 5-fold inner cross validation was used for hyperparameter tuning.

Species	Antibiotics	Isolate counts	Variant Counts	Fraction of Resistant Isolates	Accuracy
*Streptococcus pneumoniae*	tetracycline	315	1,321	6.0%	100.0%
*Streptococcus pneumoniae*	meropenem	173	1,218	5.8%	100.0%
*Streptococcus pneumoniae*	amoxicillin	171	1,208	2.3%	100.0%
*Escherichia coli*	kanamycin	75	827	13.3%	100.0%
*Staphylococcus aureus*	tetracycline	31	540	9.7%	100.0%
*Staphylococcus aureus*	clindamycin	24	479	37.5%	100.0%
*Salmonella enterica*	trimethoprim-sulfamethoxazole	1,349	1,620	0.7%	99.6%
*Streptococcus pneumoniae*	cefuroxime	178	1,221	9.6%	99.4%
*Salmonella enterica*	cefoxitin	1,291	1,483	15.9%	99.3%
*Salmonella enterica*	chloramphenicol	1,337	1,510	3.3%	99.3%
*Salmonella enterica*	amoxicillin-clavulanic acid	1,285	1,476	19.8%	99.1%
*Salmonella enterica*	kanamycin	1,036	1,373	9.4%	98.9%
*Salmonella enterica*	ceftiofur	1,340	1,489	19.0%	98.8%
*Salmonella enterica*	ceftriaxone	1,345	1,536	19.3%	98.7%
*Salmonella enterica*	tetracycline	1,339	1,518	53.0%	98.6%
*Salmonella enterica*	ampicillin	1,349	1,620	33.2%	98.5%
*Streptococcus pneumoniae*	clindamycin	316	1,323	3.5%	98.4%
*Klebsiella aerogenes*	tobramycin	58	1,014	5.2%	98.3%
*Salmonella enterica*	gentamicin	1,333	1,507	12.3%	98.0%
*Klebsiella pneumoniae*	cefotaxime	294	1,808	97.3%	97.6%
*Acinetobacter baumannii*	doripenem	204	2,027	25.0%	97.6%
*Streptococcus pneumoniae*	trimethoprim-sulfamethoxazole	275	1,143	5.8%	96.4%
*Acinetobacter baumannii*	amikacin	465	3,427	9.7%	96.4%
*Acinetobacter baumannii*	tetracycline	261	3,096	23.0%	96.2%
*Escherichia coli*	amikacin	269	1,750	6.3%	95.9%
*Enterobacter cloacae*	doripenem	44	1,167	47.7%	95.8%
*Escherichia coli*	imipenem	143	1,049	22.4%	95.8%
*Acinetobacter baumannii*	ciprofloxacin	367	3,257	73.3%	95.4%
*Streptococcus pneumoniae*	erythromycin	317	1,323	28.1%	95.3%
*Enterobacter cloacae*	tobramycin	66	1,468	39.4%	95.2%
*Escherichia coli*	ampicillin	348	2,180	92.0%	95.1%
*Klebsiella pneumoniae*	ertapenem	318	1,983	86.2%	95.0%

### Validation of the VAMPr prediction model using an external dataset

**Fig 4:**
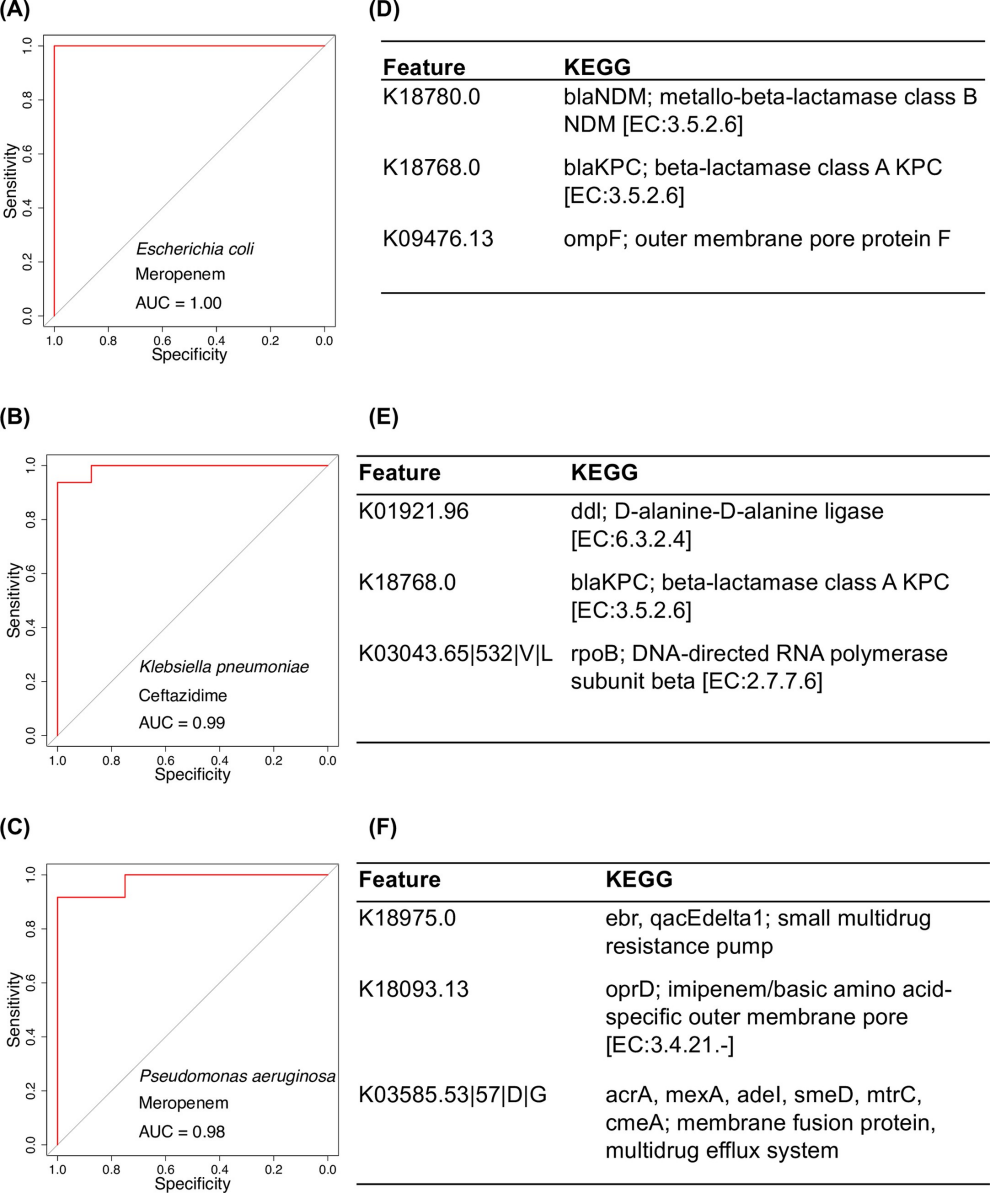
Validation performance metrics using an external dataset. AUROC (Area under the Receiver Operating Characteristic) for the prediction of the external dataset and top three predictors (KEGG orthlog variants based on importance) from the prediction models are reported. A) The AUROC curve for the *E*. *coli* and meropenem; B) The AUROC curve for the *K. pneumoniae* and ceftazidime; C) The AUROC curve for the *P*. *aeruginosa* and meropenem; D) The top three predictors for the *E*. *coli* and meropenem; E) The top three predictors for the *K. pneumoniae* and ceftazidime; F) The top three predictors for the *P*. *aeruginosa* and meropenem.

To validate the prediction performance of VAMPr, we utilized 13 *E*. *cloacae*, 31 *E*. *coli*, 24 *K*. *pneumoniae* and 21 *P*. *aeruginosa* isolates that were genetically and phenotypically profiled in a prior study but not present in the NCBI Antibiogram database [7]. All isolates had been previously tested against 3 antibiotics (cefepime, ceftazidime, and meropenem). Importantly, approximately 62%, 15%, 28% and 31% of the discovered variants of these strains, respectively, were not detected in the NCBI isolates. As these variants are specific to the validation datasets, their roles in antibiotic resistance could not be modelled by the NCBI datasets. In **Fig 4,** we show three prediction results with the highest AUROC (area under the receiver operator characteristics) values, as well as the important genetic variants that frequently appear in the gradient boosting tree models. In the *E*. *coli* and meropenem model, VAMPr reached 1.0 AUROC (**Fig 4A**) and the most important predictor was the presence of the *blaNDM* gene (New Dehli metallo-beta-lactamase; Class B). VAMPr had a similarly high prediction performance for *K*. *pneumoniae* and ceftazidime (**Fig 4B**). This model also has an AUROC value of 0.99 and the significant predictors were the presence of KPC (*K*. *pneumoniae* carbapenemase) and the presence of wildtype ddl; D-alanine-D-alanine ligase (in 4 isolates, variants of *ddl* were associated with sensitivity to ceftazidime). In **Fig 4C**, the prediction model for *P*. *aeruginosa* and meropenem is 0.95, and three significant predictors were *ebr* (small multidrug resistance pump), *mexA* (membrane fusion protein, multidrug efflux system) and *oprD* (imipenem/basic amino acid-specific outer membrane pore). Among all bacteria and antibiotic combinations, the minimal AUROC values for all VAMPr prediction models is 0.70 (**Table 2**). Additionally, we retrieved 1,688 *K*. *pneumoniae* isolates to validate the VAMPr models (**S1 Text: Validation of the VAMPr prediction model using 1,668 *K. pneumoniae* isolates**) and observed similar AUROC values. These results suggest that the VAMPr prediction models identify both known AMR-related genes as well as genes or variants that are not currently considered as contributing to resistance.

**Table 2:** External validation of VAMPr prediction model. The external dataset includes 13 *Enterobacter cloacae*, 31 *Escherichia coli*, 24 *Klebsiella pneumoniae* and 21 *Pseudomonas aeruginosa* isolates. All isolates were tested against 3 antibiotics (cefepime, ceftazidime and meropenem). We reported the accuracy as the fraction of correct predictions, and the AUROC (area under the receiver operator curve) represents the area under the operator-receiver characteristic. The AUROC value is n/a for *E*. *cloacae* as all 13 isolates are susceptible to meropenem.

Species	Antibiotics	Isolate counts	Accuracy	AUROC
*Enterobacter cloacae*	cefepime	11	100.0%	1.00
*Enterobacter cloacae*	meropenem	13	92.3%	n/a
*Escherichia coli*	cefepime	30	63.3%	0.70
*Escherichia coli*	ceftazidime	28	78.6%	0.88
*Escherichia coli*	meropenem	31	96.8%	1.00
*Klebsiella pneumoniae*	cefepime	24	70.8%	0.87
*Klebsiella pneumoniae*	ceftazidime	24	66.7%	0.99
*Klebsiella pneumoniae*	meropenem	23	78.3%	1.00
*Pseudomonas aeruginosa*	cefepime	18	83.3%	1.00
*Pseudomonas aeruginosa*	ceftazidime	21	52.4%	0.88
*Pseudomonas aeruginosa*	meropenem	20	95.0%	0.98

### Online and offline resources for VAMPr pipeline

#### Online resources-VAMPr association and prediction models

**Fig 5:**
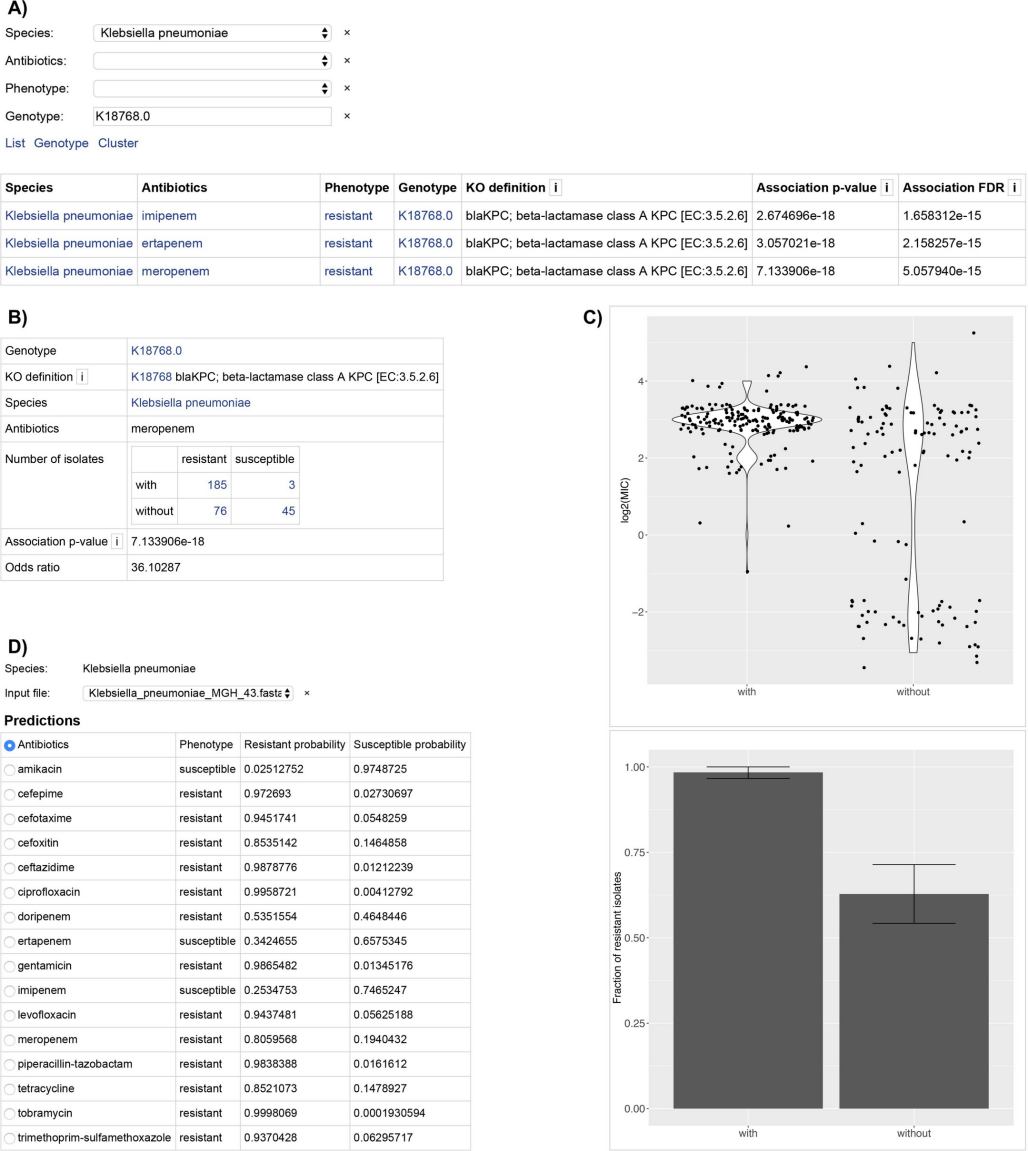
VAMPr provides rich sets of online resources for association models and prediction models. Users have the flexibility to explore known or novel antibiotic resistance-associated variants, and can upload their own sequence assembly and obtain predictions on antibiotic resistance. (A) association results webpage: users can explore variants, their interpretations, and their statistical significance assessments; (B) detailed information, contingency table and odd-ratio for variant K18768 in the association model, and distribution plots; (C) Distribution plots for variant K18768 in the association model page; (D) prediction models allow for uploads of users’ sequence data for antibiotic resistance prediction.

We provide a pre-calculated antibiotic resistance-associated variant database at https://qbrc.swmed.edu/softwares.php (**Fig 5**). Users can browse KO genetic variants and examine the strength of evidence based on calculated odds ratio and P-values from Fisher’s exact test. For prediction models, an online website and offline computational tool for users to predict antibiotic resistance from their own isolate sequences is available (see **Data Access**). The user input is the assembled FASTA files, and the online website will examine whether the sequence contains AMR genes, and if so, the exact variant of the assembled sequence. Subsequently, our prediction model will give the probability of resistance based on these sequence variants. The VAMPr model is highly efficient, as the running time of analysis is typically 60 seconds.

#### Offline resources–VAMPr source code

We provide the source code that was used to create the association and prediction models. This allows users to curate and analyze their own sequence data for convenient offline usage. For example, users can provide FASTA sequence files and predict antibiotic resistance for multiple antibiotics without an internet connection.

## Discussion

With the growing threat of antibiotic resistance and the rapidly decreasing costs associated with bacterial whole-genome sequencing, there is an opportunity for developing improved methods to detect resistance genes from genomic data [17]. However, prior to the routine use of genomic data to routinely identify bacterial AMR status, there are several hurdles to be overcome including improving our understanding of the genetic mechanisms underlying AMR for a broad-array of pathogen-antimicrobial combinations [18]. To this end, we have developed the VAMPr pipeline to discover variant-level genetic features from NGS reads which can then be correlated with phenotypic AMR data. We anticipate that with the continued generation of WGS data for numerous medically important pathogens, the widespread employment of VAMPr will assist with both strengthening associations between genomic data and AMR as well as developing new lines of AMR mechanism research.

An important advance of our study was our utilization of a novel approach to classifying variants based on gene orthologs. Our approach is different than other prediction models such as with PATRIC which utilized the adaptive boosting (adaboost) algorithm [9, 18–20]. Our results were comparable or better in performance depending on the antibiotic-pathogen combination (**S1 Text: Comparison with existing prediction models**). In addition, our approach is in contrast to other popular ways for looking at gene variants such as k-mers [21]. In the k-mer method, the frequency of k consecutive nucleotide or amino acid bases are counted as sequence features. Although the k-mer approach is straightforward to compute, it is not straightforward to explain the k-mer in the context of genes, which requires extra analysis steps to interpret. To avoid these limitations, we instead utilized gene orthologs. By aligning the bacteria genomes with a group of consensus orthologous gene sequences, we determined variants that are present for any particular AMR gene in a particular isolate. As the sequence variants are linked to ortholog genes, this approach can not only identify the presence or absence of known resistance genes, but can also give additional insight into the impact of amino acid variants on various resistance phenotypes (e.g., amino acid substitutions shown in **Fig 4**).

To understand how genetic variants were linked to AMR phenotypes, we built data-driven association models. We utilized a large collection of isolate sequence data from NCBI SRA and matching antibiotic resistance phenotypes reported in the NCBI BioSample Antibiogram. This allowed for a high throughput screening for statistically significant associations between genetic variants and specific antibiotics for a variety of pathogens. Thus, another strength of this study was the large data universe that these models were built upon with over 38,248 pathogen-antibiotic comparisons performed. Other groups have developed some similar tools, including recent efforts to predict AMR for drugs used in the treatment of *Mycobacterium tuberculosis* [22]. An advantage of VAMPr over existing tools is its ability to analyze data from any bacterial species, providing that there are sufficient numbers of bacterial genomes with AMR phenotypic data to develop robust models. The publicly available nature of VAMPr and the NCBI Antibiogram means that the predictive models of VAMPr should significantly improve moving forward.

Our attempt to develop prediction models utilizing machine learning algorithms and large-scale datasets allowed for the identification of genes that are associated with resistance to a particular antibiotic in an unbiased fashion. This could allow for both confirmation of known resistance markers as well as a discovery tool to find novel genes that contribute to resistance. It is important to note that the genes and variants that we identified as predictive of resistance does not imply causation. These are correlations, and further work will be needed to see whether identified genes that are not currently known to contribute to resistance are biologically active or just mere bystanders with other causal genes[23]. Future efforts will include testing whether some of these predicted genes or variants in genes are in fact biologically relevant. Under certain antibiotic-species combinations, the number of resistant and susceptible isolates are imbalanced. Although our current method can achieve good prediction accuracy, a specialized machine learning method for imbalanced data (e.g., SMOTE) could be employed to report model performances [24] (**S1 Text: Handling imbalanced resistant and susceptible phenotypes**).

There were other limitations of our study. Our attempt to validate the prediction models with a relatively small number of isolates that were not included in the original training set illustrates particular challenges. There was clearly strain diversity in the recently sequenced isolates that was not fully represented in the available NCBI training set which impacted our ability to fully validate our prediction models. This indicates that there continues to be a need for increased genome sequencing that is more broadly representative for certain pathogens (**S1 Text: Evaluations with additional bacterial isolates and antimicrobial susceptibility phenotypes**). This is further illustrated by the increased accuracy that was seen when we included a large number of *Klebsiella* isolates and re-ran the model. In addition, some pathogens such as *P*. *aeruginosa* have a smaller number of genomes available in the NCBI dataset with paired antibiogram data available while other pathogens (such as *Salmonella)* have a large number of genomes with AMR phenotypes available. It is likely that increasing the number of genomes available for training purposes in pathogens like *Pseudomonas* will likely further improve our accuracy of the prediction model approach (**S1 Text: Improving prediction models by augmenting external datasets**) [6]. For example, the recent study of *M*. *tuberculosis* resistance collected 10,290 samples and the large scale enabled accurate prediction of point mutations and antibiotic resistance [22]. Our future efforts are aimed at further refining the VAMPr models to include larger numbers of isolates with a mixture of antibiotic susceptibility phenotypes.

In conclusion, we are providing the VAMPr online resources for researchers to utilize in their efforts to better study and predict antibiotic resistance from bacterial whole genome sequence data. Widespread employment of VAMPr may assist with moving whole genome sequencing of bacterial pathogens out of the research lab setting and into the realm of clinical practice.

## Methods

### Data acquisition

Bacterial isolates with antibiotic susceptibility data were identified in the NCBI BioSample Antibiograms database. Isolates were identified by querying "antibiogram[filter]" in the National Center for Biotechnology Information (NCBI) (NCBI Resource Coordinators, 2018) BioSample. The linked sequencing data was downloaded from the NCBI Sequence Read Archive (SRA). Finally, the antibiogram tables in the NCBI BioSample were downloaded using NCBI API. Minimum inhibitory concentration (MIC) values and reported antibiotic susceptibility data were recorded and checked for accuracy according to CLSI guidelines [12]. MIC values that were clearly mis-annotated were removed. For the purposes of this analysis, isolates that were intermediate for any particular drug were excluded, as they only account for 0.6% of the total isolate. In addition, any bacterial isolate reported as both resistant and susceptible was excluded from analysis.

### Creation of AMR protein database

A reference database consisting of KO genes with gene-based variants was created that included both AMR protein sequences as well as AMR-like protein sequences (decoy sequences). The AMR-like sequences are from genes known to not be involved in antibiotic resistance and have been shown to improve variant calling accuracies [25]. To create the AMR protein database, a list of Kyoto Encyclopedia of Genes and Genomes (KEGG) orthology (KO) involved in antimicrobial resistance (AMR) (**S1 Fig**) was created. The protein sequences linked to AMR KOs by KEGG API and UniProt ID mapping were downloaded from the UniProt database. These sequences were designated as AMR protein sequences. Further, protein sequences from KOs not related to AMR were also aligned to AMR protein sequences. AMR-like protein sequences were defined as those protein sequences with 80% identical amino acid alignment. The union of AMR protein sequences and AMR-like protein sequences formed the AMR protein database which was utilized in all comparative alignment steps.

To facilitate the identification of variants, AMR protein sequences were clustered based on sequence identities using CD-HIT [26]. For each cluster, multiple sequence alignment (MSA) steps were used to determine cluster consensus sequences (CCS) using MAFFT [27]. Finally, bacterial isolate protein sequences were compared to CCSs to identify the variants (see next section and **S1 Text: Derive explainable KO gene-based sequence variants**).

### Characterization of AMR variants

We developed an algorithm to characterize the AMR-related variants at the protein level (**S1 Text**). For each individual bacterial isolate, *de novo* genome assembly was performed using SPAdes [28]. Open reading frame (ORF)s were identified, converted to amino acid sequences, and, protein BLAST of the sequences using the aforementioned AMR protein database was performed. The same query sequence was aligned to both AMR and AMR-like reference protein sequences using Diamond [29]. After comparative alignments and removal of less than 80% identical amino acids, only alignments best matched to the AMR reference sequences were included (see **S1 Text: Comparative alignments** for filters on E-values, bit-scores and fraction of identical amino acids and **S2 Fig**). Finally, the aligned protein scaffold sequences were compared to the CCS to define a “normal” protein versus a variant. For example, given a perfect match, an isolate is designated as carrying the KO gene and thus denoted as normal. In contrast, if there were mismatched amino acids within a CCS alignment, these would be deemed as novel variants, and in such cases, the detected variants would have the following nomenclature: KO number, KO cluster number, sequence variant types and their details (substitution, insertion, and deletion). More details are provided in **S3 Fig** and **S1 Text.**

### VAMPr association model to characterize variants

To quantitatively assess the association between KO-based sequence variants and antibiotic resistance phenotypes, an association model for each species-antibiotic combination was created. In total, 52,479 associations between variants and antibiotic resistance were evaluated. Specifically, a 2-by-2 contingency table for all isolates based on carrier/non-carrier status of the variant and susceptible/resistant phenotypes was generated and the odds ratio and p-values based on Fisher’s exact test were calculated in R 3.4.4 and adjusted for false discovery rate based on Benjamin-Hochberg procedure [30]. The fraction of resistant strains stratified by the variants’ carrying status was visualized in bar plots.

### VAMPr prediction model for antibiotic resistance

Prediction models for each species-antibiotic combination were developed. KO-based sequence variants were designated as features and curated antibiotic resistant phenotypes as labels. For each species-antibiotics combination, an optimal prediction model with tuned hyperparameters was generated. A gradient boosting tree approach was utilized, given its accurate performance profile and efficient implementation [31]. Nested cross-validation (CV) was used to report unbiased prediction performance [32, 33]. The outer CV was 10-fold and the averaged prediction metrics including accuracy are reported (**Table 1**); the inner CV was 5-fold and all inner folds were used for hyper-parameter tuning based on prediction accuracy. The default search space hyperparameters were chosen as follows: the number of rounds (the number of trees) was 50, 100, 500 or 1000; the maximum allowed depth of trees was 16 or 64; the learning rate was from 0.025 or 0.05; the minimum loss reduction required to allow further partition of the trees was 0; the fraction of features used for constructing each tree was 0.8; the fraction of isolates used for constructing each tree was 0.9; and the minimum weight for each child tree was 0. The reported performance metrics included accuracy, F1-score, and area under the receiver operating characteristic curve (AUROC) [32]. We assessed the prediction accuracy using an independent dataset of bacterial isolates recovered from cancer patients with bloodstream infections [7]. In this study (11), all isolates were genetically (whole-genome sequence) and phenotypically (antibiotic susceptibility testing by broth microdilution assays) profiled. We followed the same aforementioned genotype and phenotype processing steps. The detected KO-based variants were used as predictors and the lab-measured antibiotic resistance phenotypes were used as the gold standard. Performance metrics were calculated as described above.

### Data access

VAMPr is an open-source program. Its source codes with usage examples are available in the GitHub repository (https://github.com/jiwoongbio/VAMPr). VAMPr association model results and VAMPr online prediction models are both available from under the VAMPr link from https://qbrc.swmed.edu/softwares.php.

## Supporting information

S1 Text Supplementary texts. (PDF)

S1 Table Summary of bacterial species and antibiotic drugs combinations in association and prediction models. The resistant and susceptible isolates were counted based on the cutoff MIC (minimal inhibitory concentration) values reported in the 2018 CLSI guidelines.
(PDF)

S2 Table Bacterial isolate information. This table listed 3,393 bacterial isolates with their BioSample accession ID and antimicrobial susceptibility measurement from NCBI Antibiogram. They are included in the analysis of VAMPr association and prediction models.
(XLSX)

S3 Table A list of KEGG orthology-based antimicrobial resistant (AMR) genes. (PDF)

S4 Table Summary of prediction accuracy for 93 bacterial species and antibiotic drugs combinations using 10-fold outer cross validations. (PDF)

S1 Fig Detailed steps in VAMPr variant characterization. In VAMPr, we retrieved and curated antibiogram data from NCBI BioSample. The sequences of these isolates were retrieved from NCBI SRA, de novo assembled and curated by quality control steps (MLST identity check and phenotype QC). Based on pre-processed AMR gene databases (including both AMR protein sequences and decoy sequences), we characterize sequence variants in 9 steps, from finding gene ORF to denoting AMR gene variants based on KEGG ortholog (KO). These explainable variants, as well as the curated phenotypes, will be utilized in downstream analyses (the association models and the prediction models).
(TIF)

S2 Fig A general schematic illustration of comparative alignment. Each query protein sequence is aligned to both AMR protein sequences and decoy(AMR-like) protein sequences. We compared the best hit from the AMR protein sequences and the best hit from the decoy protein sequences. The better alignment results (denoted with “>”) based on user specified criteria (e.g. alignment scores with smaller E-values) will be retained. This step can improve alignment specificity.
(TIF)

S3 Fig Derive explainable KO gene-based sequence variants. (Upper: References (DB)) all known protein databases reference from UniProt (IDs are listed on the right); (Middle: Consensus) a consensus sequence is derived from UniProt sequences; (Bottom: Isolates) sequences from two isolates (SAMN04515808 and SAMN04254727) were compared to the consensus reference sequence, and their variants are denoted as K20319.0|94|p|I (the 94th codon of KO-gene cluster K20319.0 is changed from polar to I) and K20319.0|107|T|N (the 107th codon of KO-gene cluster K20319.0 is changed from T to N). The two variants are close, but the former variant is suggestive to induce ceftriaxone susceptibility for *A*. *baumannii* based on two isolates and the latter variant is suggestive to induce imipenem resistance based on 10 isolates.
(TIF)

S4 Fig Comparison of prediction models. We compared adaptive boosting (adaboost) [34],elastic net [16], k-nearest neighbor, 3-layer neural network (perceptron), support vector machines (with radial kernel) [35] to extreme gradient boosting tree used in VAMPr (xgboost) [31]. The boxplots show the performance difference (prediction accuracy) of xgboost to other models. All models are implemented in caret [36] and R [37]. A positive value indicates the prediction accuracy in xgboost is higher than the prediction accuracy of the other model.
(TIF)
