# Supplementary material for: VAMPr: VAriant Mapping and Prediction of antibiotic resistance via explainable features and machine learning
Source: PLoS Comput Biol. 2020 Jan 13;16(1):e1007511. doi: 10.1371/journal.pcbi.1007511 (PMC7015433; doi:10.1371/journal.pcbi.1007511)
Supplement: S1 Text — (PDF) [file pcbi.1007511.s001.pdf]

VAMPr: VAriant Mapping and Prediction of antibiotic resistance via explainable features and machine learning

Jiwoong Kim[1#], David E Greenberg[2,3#*], Reed Pifer[2], Shuang Jiang[4], Guanghua Xiao[1,5,6], Samuel A Shelburne[7], Andrew Koh[3,5,8], Yang Xie[1,5,6], and Xiaowei Zhan[1,5,9*]

[1] Quantitative Biomedical Research Center, Department of Population and Data Sciences, University of Texas Southwestern Medical Center, Dallas, Texas, 75390, USA
[2] Department of Internal Medicine, University of Texas Southwestern Medical Center, Dallas, Texas, 75390, USA
[3] Department of Microbiology, University of Texas Southwestern Medical Center, Dallas, Texas, 75390, USA
[4] Department of Statistical Science, Southern Methodist University, Dallas, TX 75275, USA
[5] Harold C. Simmons Cancer Center, University of Texas Southwestern Medical Center, Dallas, Texas, 75390, USA
[6] Department of Bioinformatics, University of Texas Southwestern Medical Center, Dallas, Texas, 75390, USA
[7] Department of Infectious Diseases and Genomic Medicine, University of Texas MD Anderson Cancer Center, Houston, Texas, USA
[8] Department of Pediatrics, University of Texas Southwestern Medical Center, Dallas, Texas, 75390, USA
[9] Center for Genetics of Host Defense, University of Texas Southwestern Medical Center, Dallas, Texas, 75390, USA

[#] The authors wish it to be known that, in their opinion, the first two authors should be regarded as joint First Authors.

* To whom correspondence should be addressed. Tel: +1-214-648-5194; Fax: +1-214-648-1663; Email: Xiaowei.Zhan@UTSouthwestern.edu, David.Greenberg@UTSouthwestern.edu

S1 Text. Supplementary Text.

Details in characterizing explainable KO-based AMR variants

A critical feature of VAMPr is to characterize explainable sequence variants based on gene ortholog. These variants have known antibiotic resistance functions. They are used in VAMPr association and prediction models. Our detailed workflow (**S6 Figure**) is as follows: 1) in the pre-processing step, we curate a list of AMR protein sequences, as well as decoy protein sequences that can reduce false positive alignments. Both aforementioned protein sequences formed the AMR protein database. 2) When VAMPr processes the user's input, e.g. assembled bacterial genomes, it searches for open-reading frames of genes, and translates the nucleotides into amino acids based on codon tables. 3) VAMPs align the input protein sequences to the reference AMR protein databases using a competitive alignment strategy, and select the best alignment hit. 4) VAMPr compares the best hit sequence to a consensus sequence, and denotes sequence variants as a protein change (e.g. amino acid substitution, insertion, and deletion).

1. Construction of AMR protein database including decoy sequences

In VAMPr, we built a reference protein database in the pre-processing step. The database was based on KEGG ortholog genes, and their sequences are retrieved from UniRef as described previously[1]. The database has two sources. The first source was the protein sequences from 537 AMR genes (**S3 Table**). The source of this gene list includes the following: a) NCBI beta-lactamase resource and Bacterial Antimicrobial Resistance Reference Gene Database (BioProject Accession Number: PRJNA313047, which initially included well-known antibiotic resistance related databases such as CARD, ResFinder et al). The protein sequences from these resources were aligned to KEGG protein sequences with protein-level BLAST with a minimum E-value of 1e-10. b) Various KEGG databases. The KEGG ortholog genes with antibiotic-resistance-related keywords from the KEGG database (KEGG BRIGHT, pathway, module, and orthology) were included. c) KEGG ortholog genes that have references, which were search results from the NCBI antibiotic-resistance-related controlled vocabulary thesaurus from PubMed. In all, based on the KEGG ortholog names, we were able to retrieve 298,760 corresponding protein sequences from UniProt (previously described[1]). The second source of the AMR protein databases was a "decoy" gene database. Basically, it was a database for genes that are not related to antibiotic resistance but have similar protein sequence contents to the first source. These protein sequences could be aligned to the AMR gene database, as any sequences were aligned with 80% or larger sequence identity to the AMR KO gene. In the end, the decoy database included 154,743 protein sequences.

2. Detection of open reading frame (ORF)

VAMPr detects the ORF from user inputted nucleotide sequences and translates them into amino acid sequences. In this step, we first *de novo* assembled the sequence reads into contigs. Then we developed a customized script to search for the start codon and stop codon based on the sequence contents. Last,

the detected ORFs were translated into amino acids based on codon tables. The translated protein sequences were used in the following alignment step.

3. Competitive alignments

VAMPr utilizes a competitive alignment strategy to achieve high alignment specificity (**S7 Figure**). In a conventional workflow, an E-value for protein BLAST needs to be pre-specified. In our workflow, we developed a data-driven approach to contrast the alignment to the AMR protein sequences to decoy protein sequences. This strategy avoids the hard-coded E-value threshold and only retains the best alignment to the AMR genes. The idea of decoy sequences has been widely used in next-generation sequence analysis [2]. In implementation, we used DIAMOND to align to the AMR protein sequences and decoy sequences, and we obtained multiple hits from the alignment outputs. If the alignment had multiple start codon positions, we only used the start codon with the shortest gene length. We filtered out the hits if the fraction of identical amino acids between query and reference was less than 80%. Next, we ranked the remaining hits based on E-values, bit-scores and fraction of identical amino acids. If the top alignment was aligned to AMR genes instead of the decoy sequences, we used this best hit in the following variant denotation step.

4. Defining KO-based AMR variants

VAMPr compares the best hit alignment to the reference consensus protein sequence to denote KO-based AMR variants. As illustrated in **S8 Figure**, VAMPr first aligned all AMR gene protein sequences from the UniPort database and reduced the number of protein sequences to 96,462 protein clusters based on a sequence identity of 70% or higher using CD-Hit. These sequences then formed a consensus reference sequence to which all query sequences can be locally aligned using MAFFT[3]. We denoted the consensus amino acid using 23 amino acid letters, or denoted the conserved basic, acidic, polar, and hydrophobic residue amino as "b", "a", "p", and "h", respectively. The random and gap residues were denoted by "." and "_". The preparation of these consensus sequences only needs to be performed once. Then VAMPr reports the alignment of the query protein sequence variant. For example (**S8 Figure)**, the genome of isolate SAMN04515808 was assembled and aligned to KO cluster K20319.0 (blaADC; beta-lactamase class C ADC [EC:3.5.2.6]). VAMPr discovered that the 94th codon was changed from p to I, which may contribute to the acquired ceftriaxone susceptibility. Similarly, the genome of isolate SAMN04254727 was assembled and aligned to the same KO cluster and VAMPr discovered its 107th codon seems to induce imipenem resistance based on 10 isolates. This showed that two close variants from the same gene may lead to different antibiotic resistance phenotypes. Therefore, these explainable KO gene-based variants calculated by VAMPr offered useful information in addition to the detection of existence of AMR genes.

Comparison with existing prediction models

PATRIC is popular prediction model for antibiotic resistance. Based on its model release page (https://github.com/PATRIC3/mic_prediction), we found the release version includes a *K. pneumoniae* prediction model. We compared its prediction performance based on the combination of three antibiotics and 24 in-house *K. pneumoniae* isolates . As PATRIC predictions were MIC values, it is natural to compare them to the observed MIC values. We found only 7% of the predicted MIC values were correct. Then we followed CLSI guidelines to classify the MIC predictions as resistant or susceptible [4]. The accuracy for the PATRIC model is 88.7%. Finally, we used the existing VAMPr model to predict antibiotic resistance, and we used the cutoff values based on the ratio of resistant isolates. The prediction accuracy is also 88.7%. When we adjusted for the imbalanced class using the SMOTE method [5], the VAMPr model can achieve 91.5% accuracy. The above analysis shows that the VAMPr prediction model has similar or better prediction accuracy compared to PATRIC in *K. pneumoniae* and cefepime/ceftazidime/meropenem combinations.

Improving prediction models by augmenting external datasets

When the training dataset includes more bacteria and antibiotic combinations, we hypothesize that the prediction model will have better accuracy. Thus we incorporated 1,668 *K. pneumoniae* isolates by Nguyen [6] in addition to the 344 isolates curated from NCBI Antibiogram. For all the isolates, the antimicrobial tests were performed for the following 8 antibiotics: aztreonam, cefazolin, cefepime, ceftazidime, ceftriaxone, imipenem, meropenem, and nitrofurantoin. We utilized the same way to construct the prediction model so the change of accuracy will reflect the effect of a larger dataset. Our original model (trained on 344 isolates) achieved 90.6% accuracy. The new model (trained on the combination of 344 and 1,688 isolates) achieved 92.1% accuracy. We further compared the prediction performance using an independent dataset of 24 *K. pneumoniae isolates [7]*. The average prediction accuracy is 84.5% (the original model) and 88.8% (the new model). These observations confirmed that a large collection of *K. pneumoniae*-antibiotic combinations can improve the prediction accuracies.

Validation of the VAMPr prediction model using 1,668 *K. Pneumoniae* isolates

We performed the validation of VAMPr prediction mode by using the 1,668 *K. pneumoniae* isolates published by Nguyen [6] in addition to the 24 isolates published by us *[7]*. There are three antibiotics tested for both datasets: cefepime, ceftazidime, and meropenem. The prediction accuracy using the original 24 isolates for these antibiotics are: 70.8%, 66.7%, and 78.3%, respectively. The prediction accuracy using the 1,668 isolates for the these antibiotics are: 71.5%, 91.7%, and 65.3% respectively. These results demonstrated that the prediction performance for cefepime are similar for both datasets. Meanwhile, in the large Nguyen's datasets, the accuracy is higher for ceftazidime and lower for meropenem.

Handling imbalanced resistant and susceptible phenotypes

The NCBI Antibiogram includes bacterial-antibiotic combinations where the number of resistant isolates and susceptible isolates are imbalanced. Here we wanted to quantitatively assess its impact on VAMPrs model performance. We applied a popular machine learning algorithm, SMOTE [5], to synthesize minority samples, and constructed the prediction models following the same procedure. Across the 93 prediction models, the average accuracy is 91.9% for the original VAMPr models and is 89.7% for the SMOTE models. Then we evaluated the model performances using an independent dataset of 89 isolates *[7]*. We reported the AUROC for the original VAMPr model to be: 1.00 (*E. coli* and meropenem), 1.00 (*K. pneumoniae* and ceftazidime) and 0.93 (*P. aeruginosa* and meropenem), while the AUROC for the SMOTE model is 1.00, 0.95, and 0.94 respectively. Overall, similar performance was observed between the SMOTE model and the original model.

REFERENCES

1. Kim J, Kim MS, Koh AY, Xie Y, Zhan X. FMAP: Functional Mapping and Analysis Pipeline for metagenomics and metatranscriptomics studies. BMC Bioinformatics. 2016;17(1):420. Epub 2016/10/12. doi: 10.1186/s12859-016-1278-0. PubMed PMID: 27724866; PubMed Central PMCID: PMCPMC5057277.
2. Buchfink B, Xie C, Huson DH. Fast and sensitive protein alignment using DIAMOND. Nature methods. 2015;12(1):59-60. Epub 2014/11/18. doi: 10.1038/nmeth.3176. PubMed PMID: 25402007.
3. Nakamura T, Yamada KD, Tomii K, Katoh K. Parallelization of MAFFT for large-scale multiple sequence alignments. Bioinformatics. 2018;34(14):2490-2. Epub 2018/03/06. doi: 10.1093/bioinformatics/bty121. PubMed PMID: 29506019; PubMed Central PMCID: PMCPMC6041967.
4. CLSI. Performance Standards for Antimicrobial Susceptibility Testing. 28th ed. CLSI supplement M100 ed. Wayne, PA: Clinical and Laboratory Standards Institute2018 2018.
5. Chawla NV, Bowyer KW, Hall LO, Kegelmeyer WP. SMOTE: synthetic minority over-sampling technique. Journal of artificial intelligence research. 2002;16:321-57.
6. Nguyen M, Brettin T, Long SW, Musser JM, Olsen RJ, Olson R, et al. Developing an in silico minimum inhibitory concentration panel test for Klebsiella pneumoniae. Sci Rep. 2018;8(1):421. Epub 2018/01/13. doi: 10.1038/s41598-017-18972-w. PubMed PMID: 29323230; PubMed Central PMCID: PMCPMC5765115.
7. Shelburne SA, Kim J, Munita JM, Sahasrabhojane P, Shields RK, Press EG, et al. Whole-Genome Sequencing Accurately Identifies Resistance to Extended-Spectrum β-Lactams for Major Gram-Negative Bacterial Pathogens. Clinical Infectious Diseases. 2017;65(5):738-45. Epub 2017/05/05. doi: 10.1093/cid/cix417. PubMed PMID: 28472260; PubMed Central PMCID: PMCPMC5850535.
