# Supplementary material for: VAMPr: VAriant Mapping and Prediction of antibiotic resistance via explainable features and machine learning
Source: PLoS Comput Biol. 2020 Jan 13;16(1):e1007511. doi: 10.1371/journal.pcbi.1007511 (PMC7015433; doi:10.1371/journal.pcbi.1007511)
Supplement: S1 Table — The resistant and susceptible isolates were counted based on the cutoff MIC (minimal inhibitory concentration) values reported in the 2018 CLSI guidelines. (PDF) [file pcbi.1007511.s002.pdf]

**S1 Table. Summary of bacterial species and antibiotic drugs combinations in association and prediction models.** The resistant and susceptible isolates were counted based on the cutoff MIC (minimal inhibitory concentration) values reported in the 2018 CLSI guidelines.

| Species | Antibiotics | Resistant Isolates | Susceptible Isolates | Total Isolates | Percentage of Resistant Isolates |
|---|---|---|---|---|---|
| *Acinetobacter baumannii* | amikacin | 45 | 420 | 465 | 9.7% |
| *Acinetobacter baumannii* | ampicillin-sulbactam | 307 | 389 | 696 | 44.1% |
| *Acinetobacter baumannii* | cefotaxime | 53 | 25 | 78 | 67.9% |
| *Acinetobacter baumannii* | ciprofloxacin | 269 | 98 | 367 | 73.3% |
| *Acinetobacter baumannii* | doripenem | 51 | 153 | 204 | 25.0% |
| *Acinetobacter baumannii* | levofloxacin | 171 | 151 | 322 | 53.1% |
| *Acinetobacter baumannii* | meropenem | 170 | 136 | 306 | 55.6% |
| *Acinetobacter baumannii* | tetracycline | 60 | 201 | 261 | 23.0% |
| *Acinetobacter baumannii* | tobramycin | 100 | 343 | 443 | 22.6% |
| *Enterobacter cloacae* | cefepime | 37 | 19 | 56 | 66.1% |
| *Enterobacter cloacae* | ciprofloxacin | 40 | 35 | 75 | 53.3% |
| *Enterobacter cloacae* | doripenem | 21 | 23 | 44 | 47.7% |
| *Enterobacter cloacae* | ertapenem | 39 | 20 | 59 | 66.1% |
| *Enterobacter cloacae* | gentamicin | 13 | 61 | 74 | 17.6% |
| *Enterobacter cloacae* | imipenem | 28 | 26 | 54 | 51.9% |
| *Enterobacter cloacae* | levofloxacin | 34 | 34 | 68 | 50.0% |
| *Enterobacter cloacae* | meropenem | 31 | 36 | 67 | 46.3% |
| *Enterobacter cloacae* | tetracycline | 19 | 36 | 55 | 34.5% |
| *Enterobacter cloacae* | tobramycin | 26 | 40 | 66 | 39.4% |
| *Enterobacter cloacae* | trimethoprim-sulfamethoxazole | 38 | 33 | 71 | 53.5% |
| *Escherichia coli* | amikacin | 17 | 252 | 269 | 6.3% |
| *Escherichia coli* | amoxicillin-clavulanic acid | 135 | 128 | 263 | 51.3% |
| *Escherichia coli* | ampicillin | 320 | 28 | 348 | 92.0% |
| *Escherichia coli* | aztreonam | 122 | 29 | 151 | 80.8% |

| | | | | | |
|---|---|---|---|---|---|
| *Escherichia coli* | cefazolin | 110 | 13 | 123 | 89.4% |
| *Escherichia coli* | cefepime | 107 | 108 | 215 | 49.8% |
| *Escherichia coli* | cefoxitin | 90 | 139 | 229 | 39.3% |
| *Escherichia coli* | ceftazidime | 152 | 59 | 211 | 72.0% |
| *Escherichia coli* | ceftiofur | 23 | 53 | 76 | 30.3% |
| *Escherichia coli* | ceftriaxone | 267 | 78 | 345 | 77.4% |
| *Escherichia coli* | chloramphenicol | 55 | 21 | 76 | 72.4% |
| *Escherichia coli* | ciprofloxacin | 216 | 131 | 347 | 62.2% |
| *Escherichia coli* | doripenem | 24 | 64 | 88 | 27.3% |
| *Escherichia coli* | ertapenem | 55 | 202 | 257 | 21.4% |
| *Escherichia coli* | gentamicin | 100 | 240 | 340 | 29.4% |
| *Escherichia coli* | imipenem | 32 | 111 | 143 | 22.4% |
| *Escherichia coli* | kanamycin | 10 | 65 | 75 | 13.3% |
| *Escherichia coli* | levofloxacin | 206 | 60 | 266 | 77.4% |
| *Escherichia coli* | meropenem | 38 | 228 | 266 | 14.3% |
| *Escherichia coli* | piperacillin-tazobactam | 66 | 174 | 240 | 27.5% |
| *Escherichia coli* | tetracycline | 209 | 102 | 311 | 67.2% |
| *Escherichia coli* | tobramycin | 76 | 117 | 193 | 39.4% |
| *Escherichia coli* | trimethoprim-sulfamethoxazole | 187 | 161 | 348 | 53.7% |
| *Klebsiella aerogenes* | cefepime | 7 | 44 | 51 | 13.7% |
| *Klebsiella aerogenes* | ceftazidime | 55 | 9 | 64 | 85.9% |
| *Klebsiella aerogenes* | tobramycin | 3 | 55 | 58 | 5.2% |
| *Klebsiella aerogenes* | trimethoprim-sulfamethoxazole | 6 | 54 | 60 | 10.0% |
| *Klebsiella pneumoniae* | amikacin | 61 | 204 | 265 | 23.0% |
| *Klebsiella pneumoniae* | cefepime | 249 | 34 | 283 | 88.0% |
| *Klebsiella pneumoniae* | cefotaxime | 286 | 8 | 294 | 97.3% |
| *Klebsiella pneumoniae* | cefoxitin | 160 | 38 | 198 | 80.8% |
| *Klebsiella pneumoniae* | ceftazidime | 298 | 15 | 313 | 95.2% |
| *Klebsiella pneumoniae* | ciprofloxacin | 243 | 36 | 279 | 87.1% |

| | | | | | |
|---|---|---|---|---|---|
| *Klebsiella pneumoniae* | doripenem | 174 | 26 | 200 | 87.0% |
| *Klebsiella pneumoniae* | ertapenem | 274 | 44 | 318 | 86.2% |
| *Klebsiella pneumoniae* | gentamicin | 129 | 166 | 295 | 43.7% |
| *Klebsiella pneumoniae* | imipenem | 247 | 33 | 280 | 88.2% |
| *Klebsiella pneumoniae* | levofloxacin | 271 | 37 | 308 | 88.0% |
| *Klebsiella pneumoniae* | meropenem | 261 | 48 | 309 | 84.5% |
| *Klebsiella pneumoniae* | piperacillin-tazobactam | 202 | 19 | 221 | 91.4% |
| *Klebsiella pneumoniae* | tetracycline | 117 | 103 | 220 | 53.2% |
| *Klebsiella pneumoniae* | tobramycin | 201 | 47 | 248 | 81.0% |
| *Klebsiella pneumoniae* | trimethoprim-sulfamethoxazole | 254 | 67 | 321 | 79.1% |
| *Pseudomonas aeruginosa* | amikacin | 23 | 53 | 76 | 30.3% |
| *Pseudomonas aeruginosa* | aztreonam | 42 | 31 | 73 | 57.5% |
| *Pseudomonas aeruginosa* | cefepime | 49 | 24 | 73 | 67.1% |
| *Pseudomonas aeruginosa* | ceftazidime | 52 | 25 | 77 | 67.5% |
| *Pseudomonas aeruginosa* | doripenem | 54 | 22 | 76 | 71.1% |
| *Pseudomonas aeruginosa* | gentamicin | 45 | 31 | 76 | 59.2% |
| *Pseudomonas aeruginosa* | imipenem | 68 | 12 | 80 | 85.0% |
| *Pseudomonas aeruginosa* | meropenem | 56 | 17 | 73 | 76.7% |
| *Pseudomonas aeruginosa* | piperacillin-tazobactam | 44 | 27 | 71 | 62.0% |
| *Pseudomonas aeruginosa* | tobramycin | 47 | 34 | 81 | 58.0% |
| *Salmonella enterica* | amoxicillin-clavulanic acid | 254 | 1031 | 1,285 | 19.8% |
| *Salmonella enterica* | ampicillin | 448 | 901 | 1,349 | 33.2% |
| *Salmonella enterica* | cefoxitin | 205 | 1086 | 1,291 | 15.9% |
| *Salmonella enterica* | ceftiofur | 254 | 1086 | 1,340 | 19.0% |
| *Salmonella enterica* | ceftriaxone | 260 | 1085 | 1,345 | 19.3% |
| *Salmonella enterica* | chloramphenicol | 44 | 1293 | 1,337 | 3.3% |
| *Salmonella enterica* | gentamicin | 164 | 1169 | 1,333 | 12.3% |
| *Salmonella enterica* | kanamycin | 97 | 939 | 1,036 | 9.4% |
| *Salmonella enterica* | tetracycline | 710 | 629 | 1,339 | 53.0% |

| | | | | | |
|---|---|---|---|---|---|
| *Salmonella enterica* | trimethoprim-sulfamethoxazole | 9 | 1340 | 1,349 | 0.7% |
| *Staphylococcus aureus* | clindamycin | 9 | 15 | 24 | 37.5% |
| *Staphylococcus aureus* | levofloxacin | 21 | 10 | 31 | 67.7% |
| *Staphylococcus aureus* | tetracycline | 3 | 28 | 31 | 9.7% |
| *Streptococcus pneumoniae* | amoxicillin | 4 | 167 | 171 | 2.3% |
| *Streptococcus pneumoniae* | cefuroxime | 17 | 161 | 178 | 9.6% |
| *Streptococcus pneumoniae* | clindamycin | 11 | 305 | 316 | 3.5% |
| *Streptococcus pneumoniae* | erythromycin | 89 | 228 | 317 | 28.1% |
| *Streptococcus pneumoniae* | meropenem | 10 | 163 | 173 | 5.8% |
| *Streptococcus pneumoniae* | tetracycline | 19 | 296 | 315 | 6.0% |
| *Streptococcus pneumoniae* | trimethoprim-sulfamethoxazole | 16 | 259 | 275 | 5.8% |
