# Supplementary material for: VAMPr: VAriant Mapping and Prediction of antibiotic resistance via explainable features and machine learning
Source: PLoS Comput Biol. 2020 Jan 13;16(1):e1007511. doi: 10.1371/journal.pcbi.1007511 (PMC7015433; doi:10.1371/journal.pcbi.1007511)
Supplement: S3 Table — (PDF) [file pcbi.1007511.s004.pdf]

S3 Table. A list of KEGG orthology-based antimicrobial resistant (AMR) genes.

Evidence	KEGG orthology
(Protein BLAST) NCBI Beta-lactamase resources[+]	56
(Protein BLAST) PRJNA313047: Bacterial Antimicrobial Resistance Reference Gene Database	187
KEGG BRITE: Antimicrobial resistance	237
KEGG pathway: beta-Lactam resistance, Vancomycin resistance, CAMP resistance, Penicillin	184
KEGG module: penicillin-binding protein, multidrug efflux system, antibiotic resistance	212
KEGG orthology: penicillin-binding protein, multidrug efflux system, antibiotic resistance	179
PubMed MeSH: "Drug Resistance, Bacterial", "beta-lactamases" reported in KEGG	202
**Total**	**537**

+: NCBI Beta-lactamase resources https://www.ncbi.nlm.nih.gov/pathogens/beta-lactamase-data-resources/

*: PRJNA313047: Bacterial Antimicrobial Resistance Reference Gene Database https://www.ncbi.nlm.nih.gov/bioproject/PRJNA313047

This resource includes the following: CARD[8], Lahey, Pasteur Institute Beta Lactamases, ResFinder[9], NCBI AMR FTP, NCBI AMR HMMs.
