# Supplementary material for: VAMPr: VAriant Mapping and Prediction of antibiotic resistance via explainable features and machine learning
Source: PLoS Comput Biol. 2020 Jan 13;16(1):e1007511. doi: 10.1371/journal.pcbi.1007511 (PMC7015433; doi:10.1371/journal.pcbi.1007511)
Supplement: S4 Table — (PDF) [file pcbi.1007511.s005.pdf]

S4 Table. Summary of prediction accuracy for 93 bacterial species and antibiotic drugs combinations using 10-fold outer cross validations.

Species	Antibiotics	Prediction Accuracy
Acinetobacter baumannii	amikacin	96.4%
Acinetobacter baumannii	ampicillin-sulbactam	94.8%
Acinetobacter baumannii	cefotaxime	91.0%
Acinetobacter baumannii	ciprofloxacin	95.4%
Acinetobacter baumannii	doripenem	97.6%
Acinetobacter baumannii	levofloxacin	93.5%
Acinetobacter baumannii	meropenem	94.8%
Acinetobacter baumannii	tetracycline	96.2%
Acinetobacter baumannii	tobramycin	94.8%
Enterobacter cloacae	cefepime	85.2%
Enterobacter cloacae	ciprofloxacin	86.8%
Enterobacter cloacae	doripenem	95.8%
Enterobacter cloacae	ertapenem	81.0%
Enterobacter cloacae	gentamicin	89.5%
Enterobacter cloacae	imipenem	78.3%
Enterobacter cloacae	levofloxacin	85.2%
Enterobacter cloacae	meropenem	79.0%
Enterobacter cloacae	tetracycline	76.0%
Enterobacter cloacae	tobramycin	95.2%
Enterobacter cloacae	trimethoprim-sulfamethoxazole	94.5%
Escherichia coli	amikacin	95.9%
Escherichia coli	amoxicillin-clavulanic acid	92.8%
Escherichia coli	ampicillin	95.1%
Escherichia coli	aztreonam	87.9%
Escherichia coli	cefazolin	88.8%
Escherichia coli	cefepime	77.7%
Escherichia coli	cefoxitin	89.1%
Escherichia coli	ceftazidime	77.8%
Escherichia coli	ceftiofur	93.7%
Escherichia coli	ceftriaxone	90.4%
Escherichia coli	chloramphenicol	90.9%
Escherichia coli	ciprofloxacin	89.1%
Escherichia coli	doripenem	78.9%
Escherichia coli	ertapenem	91.5%

Escherichia coli	gentamicin	94.7%
Escherichia coli	imipenem	95.8%
Escherichia coli	kanamycin	100.0%
Escherichia coli	levofloxacin	87.6%
Escherichia coli	meropenem	91.3%
Escherichia coli	piperacillin-tazobactam	90.3%
Escherichia coli	tetracycline	94.2%
Escherichia coli	tobramycin	93.3%
Escherichia coli	trimethoprim-sulfamethoxazole	93.7%
Klebsiella aerogenes	cefepime	92.3%
Klebsiella aerogenes	ceftazidime	90.5%
Klebsiella aerogenes	tobramycin	98.3%
Klebsiella aerogenes	trimethoprim-sulfamethoxazole	93.3%
Klebsiella pneumoniae	amikacin	87.6%
Klebsiella pneumoniae	cefepime	92.9%
Klebsiella pneumoniae	cefotaxime	97.6%
Klebsiella pneumoniae	cefoxitin	84.8%
Klebsiella pneumoniae	ceftazidime	94.9%
Klebsiella pneumoniae	ciprofloxacin	91.0%
Klebsiella pneumoniae	doripenem	87.0%
Klebsiella pneumoniae	ertapenem	95.0%
Klebsiella pneumoniae	gentamicin	93.6%
Klebsiella pneumoniae	imipenem	90.7%
Klebsiella pneumoniae	levofloxacin	92.2%
Klebsiella pneumoniae	meropenem	90.9%
Klebsiella pneumoniae	piperacillin-tazobactam	90.9%
Klebsiella pneumoniae	tetracycline	78.6%
Klebsiella pneumoniae	tobramycin	90.7%
Klebsiella pneumoniae	trimethoprim-sulfamethoxazole	89.7%
Pseudomonas aeruginosa	amikacin	83.4%
Pseudomonas aeruginosa	aztreonam	69.6%
Pseudomonas aeruginosa	cefepime	78.2%
Pseudomonas aeruginosa	ceftazidime	75.1%
Pseudomonas aeruginosa	doripenem	78.4%
Pseudomonas aeruginosa	gentamicin	81.1%
Pseudomonas aeruginosa	imipenem	87.6%
Pseudomonas aeruginosa	meropenem	79.8%
Pseudomonas aeruginosa	piperacillin-tazobactam	80.1%
Pseudomonas aeruginosa	tobramycin	85.3%

Salmonella enterica	amoxicillin-clavulanic acid	99.1%
Salmonella enterica	ampicillin	98.5%
Salmonella enterica	cefoxitin	99.3%
Salmonella enterica	ceftiofur	98.8%
Salmonella enterica	ceftriaxone	98.7%
Salmonella enterica	chloramphenicol	99.3%
Salmonella enterica	gentamicin	98.0%
Salmonella enterica	kanamycin	98.9%
Salmonella enterica	tetracycline	98.6%
Salmonella enterica	trimethoprim-sulfamethoxazole	99.6%
Staphylococcus aureus	clindamycin	100.0%
Staphylococcus aureus	levofloxacin	90.0%
Staphylococcus aureus	tetracycline	100.0%
Streptococcus pneumoniae	amoxicillin	100.0%
Streptococcus pneumoniae	cefuroxime	99.4%
Streptococcus pneumoniae	clindamycin	98.4%
Streptococcus pneumoniae	erythromycin	95.3%
Streptococcus pneumoniae	meropenem	100.0%
Streptococcus pneumoniae	tetracycline	100.0%
Streptococcus pneumoniae	trimethoprim-sulfamethoxazole	96.4%
