# Supplementary figures and images for: VAMPr: VAriant Mapping and Prediction of antibiotic resistance via explainable features and machine learning
Source: PLoS Comput Biol. 2020 Jan 13;16(1):e1007511. doi: 10.1371/journal.pcbi.1007511 (PMC7015433; doi:10.1371/journal.pcbi.1007511)

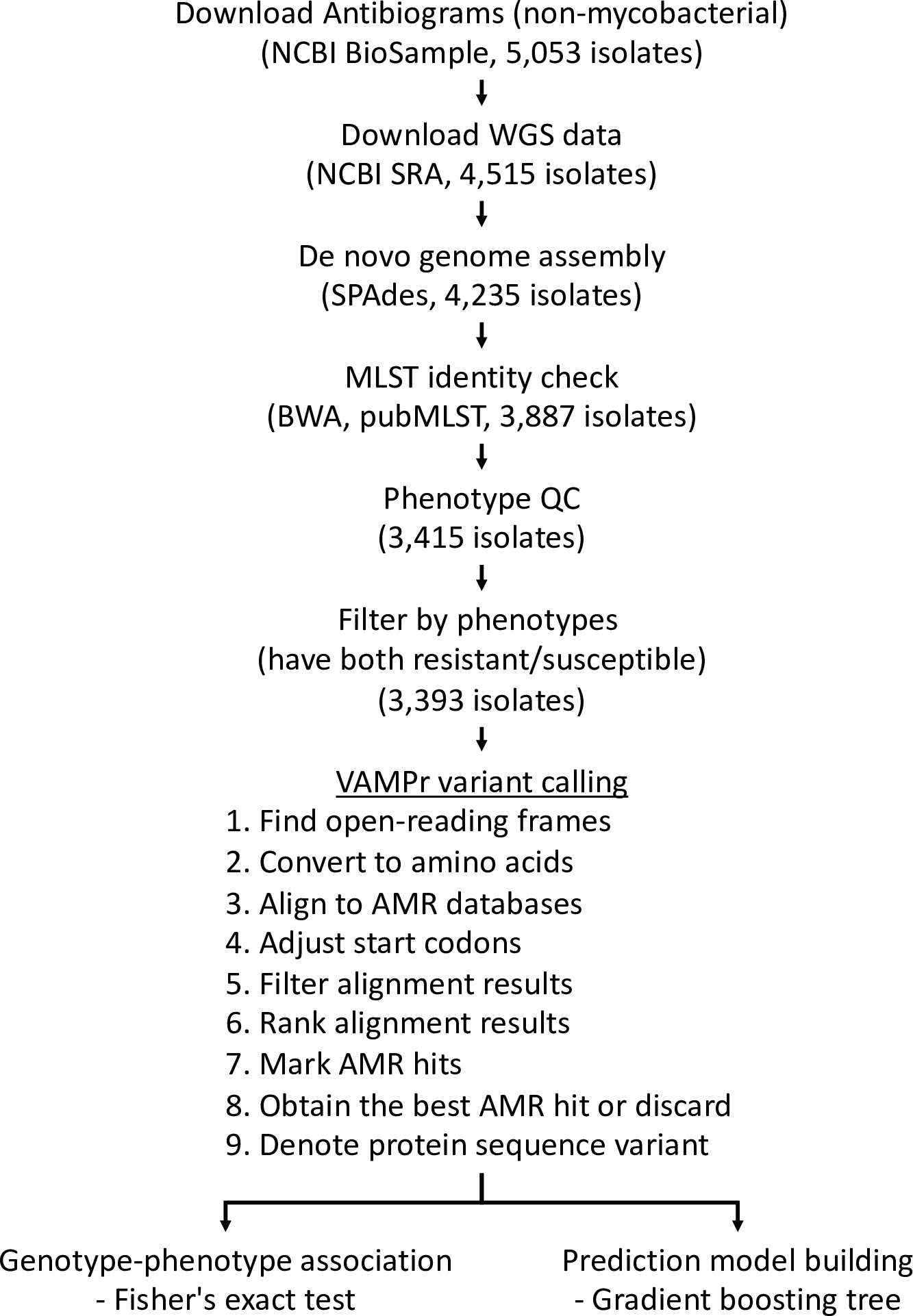

Supplement: S1 Fig — In VAMPr, we retrieved and curated antibiogram data from NCBI BioSample. The sequences of these isolates were retrieved from NCBI SRA, de novo assembled and curated by quality control steps (MLST identity check and phenotype QC). Based on pre-processed AMR gene databases (including both AMR protein sequences and decoy sequences), we characterize sequence variants in 9 steps, from finding gene ORF to denoting AMR gene variants based on KEGG ortholog (KO). These explainable variants, as well as the curated phenotypes, will be utilized in downstream analyses (the association models and the prediction models). (TIF) [file pcbi.1007511.s006.tif]

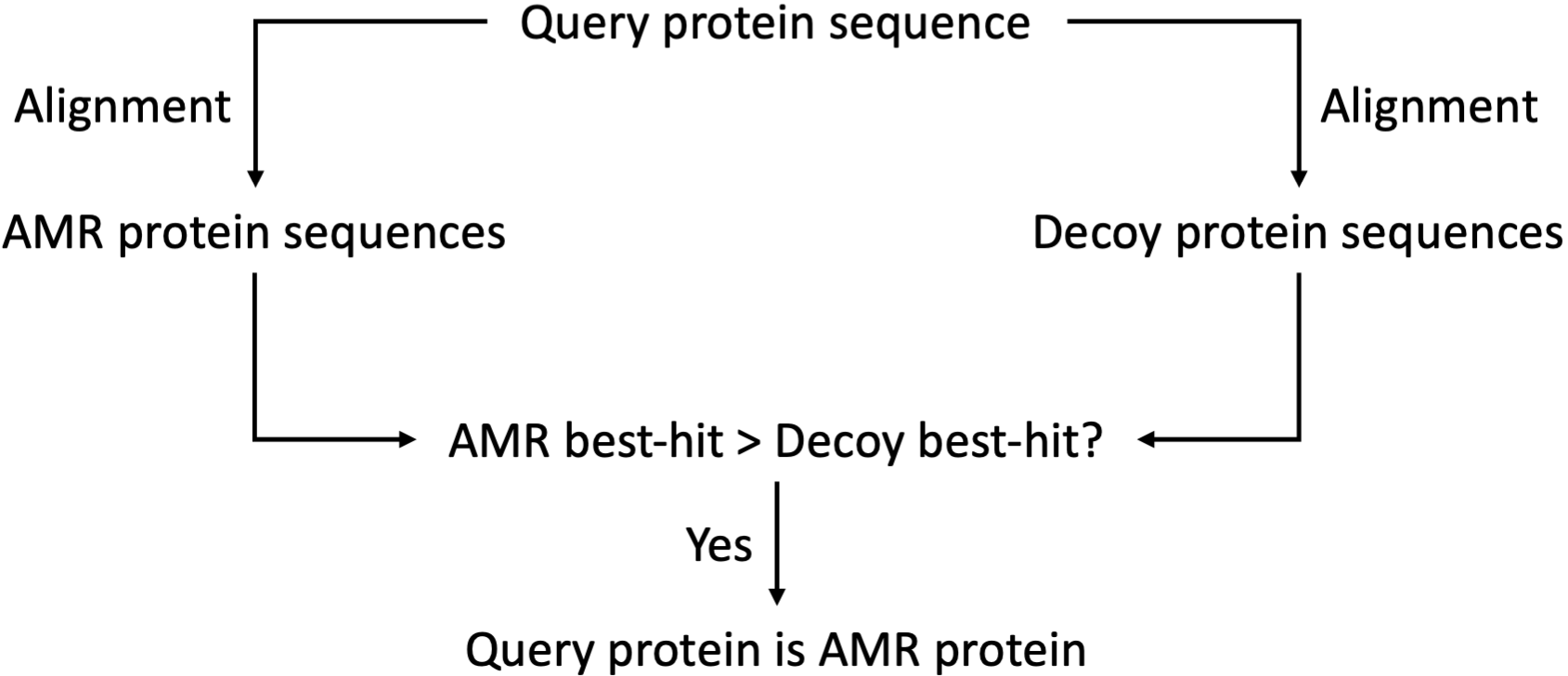

Supplement: S2 Fig — Each query protein sequence is aligned to both AMR protein sequences and decoy(AMR-like) protein sequences. We compared the best hit from the AMR protein sequences and the best hit from the decoy protein sequences. The better alignment results (denoted with “>”) based on user specified criteria (e.g. alignment scores with smaller E-values) will be retained. This step can improve alignment specificity. (TIF) [file pcbi.1007511.s007.tif]

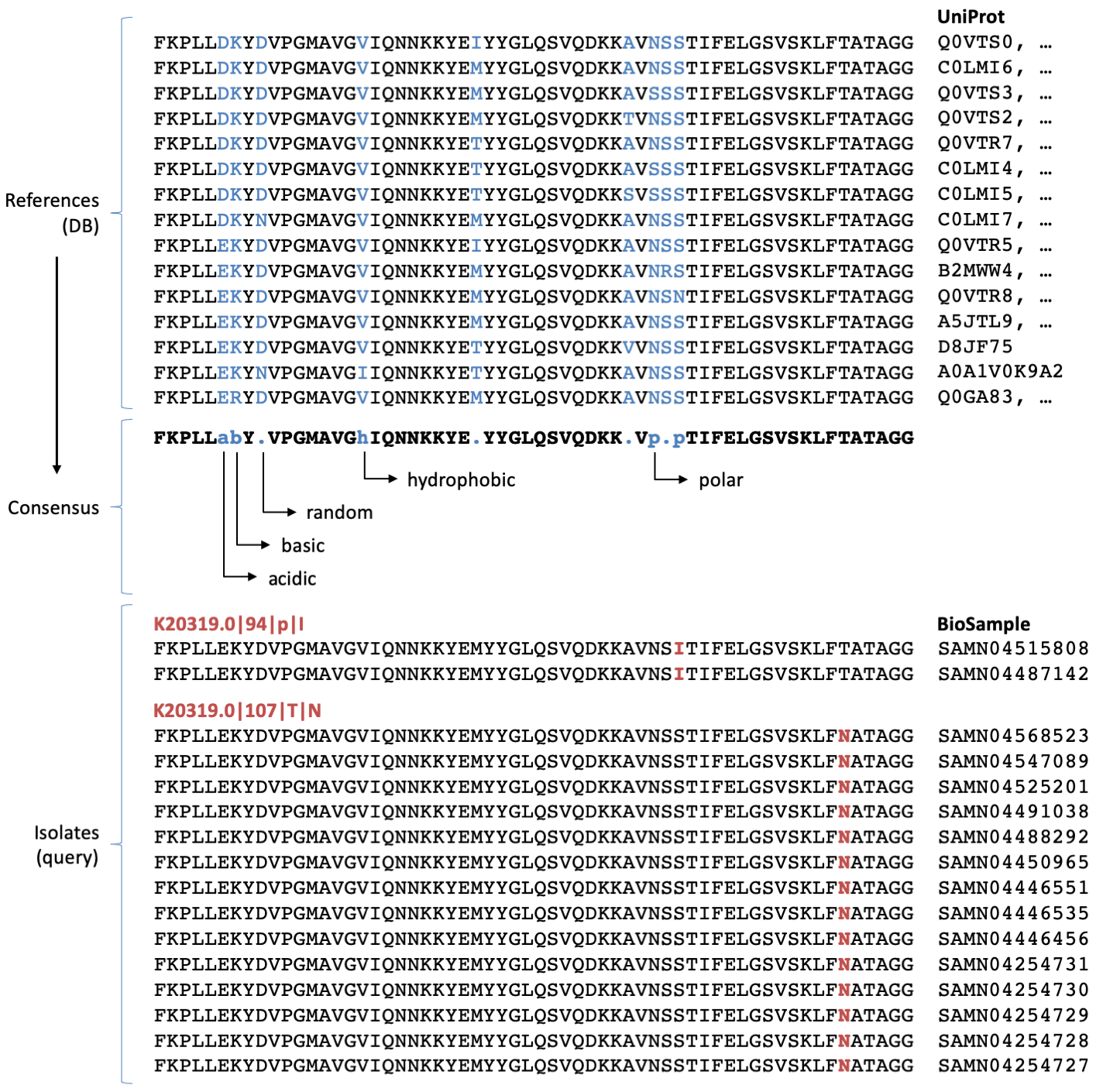

Supplement: S3 Fig — (Upper: References (DB)) all known protein databases reference from UniProt (IDs are listed on the right); (Middle: Consensus) a consensus sequence is derived from UniProt sequences; (Bottom: Isolates) sequences from two isolates (SAMN04515808 and SAMN04254727) were compared to the consensus reference sequence, and their variants are denoted as K20319.0|94|p|I (the 94th codon of KO-gene cluster K20319.0 is changed from polar to I) and K20319.0|107|T|N (the 107th codon of KO-gene cluster K20319.0 is changed from T to N). The two variants are close, but the former variant is suggestive to induce ceftriaxone susceptibility for A. baumannii based on two isolates and the latter variant is suggestive to induce imipenem resistance based on 10 isolates. (TIF) [file pcbi.1007511.s008.tif]

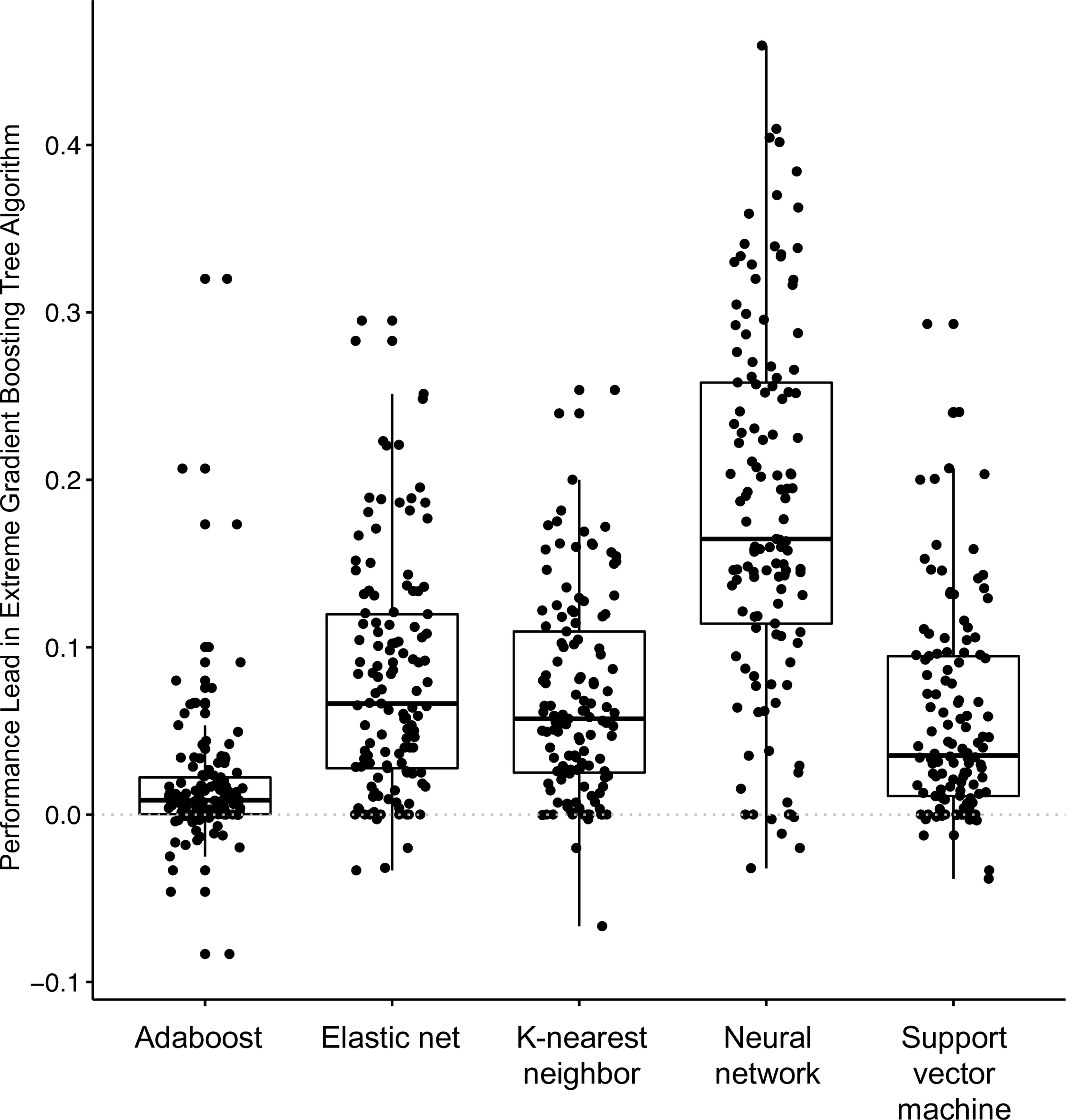

Supplement: S4 Fig — We compared adaptive boosting (adaboost) [34],elastic net [16], k-nearest neighbor, 3-layer neural network (perceptron), support vector machines (with radial kernel) [35] to extreme gradient boosting tree used in VAMPr (xgboost) [31]. The boxplots show the performance difference (prediction accuracy) of xgboost to other models. All models are implemented in caret [36] and R [37]. A positive value indicates the prediction accuracy in xgboost is higher than the prediction accuracy of the other model. (TIF) [file pcbi.1007511.s009.tif]
